# A Survey of Peptides with Effective Therapeutic Potential in Alzheimer’s Disease Rodent Models or in Human Clinical Studies

**DOI:** 10.2174/138955712800493942

**Published:** 2012-05

**Authors:** N Sun, SA Funke, D Willbold

**Affiliations:** 1 ICS-6, Forschungszentrum Jülich, 52425 Jülich, Germany; 2 Institut für Physikalische Biologie, Heinrich-Heine- Universität, 40225 Düsseldorf, Germany

**Keywords:** Alzheimer’s disease, amyloid-β, D-enantiomer, mirror image phage display, therapeutic peptide, β-sheet breaker.

## Abstract

Alzheimer’s disease (AD) is a devastating neurodegenerative disorder and the most common cause of dementia. Today, only palliative therapies are available. The pathological hallmarks of AD are the presence of neurofibrillary tangles and amyloid plaques, mainly composed of the amyloid-β peptide (Aβ), in the brains of the patients. Several lines of evidence suggest that the increased production and/or decreased cleavage of Aβ and subsequent accumulation of Aβ oligomers and aggregates play a fundamental role in the disease progress. Therefore, substances which bind to Aβ and influence aggregation thereof are of great interest. A wide range of Aβ binding peptides were investigated to date for therapeutic purposes. Only very few were shown to be effective in rodent AD models or in clinical studies. Here, we review those peptides and discuss their possible mechanisms of action.

## INTRODUCTION

Alzheimer’s disease (AD) is a progressive neurodegen-erative disorder and the foremost cause of dementia in elderly people [1]. There are more than 20 million sufferers worldwide. AD is predicted to affect 1 in 85 people globally by 2050 [2].

Extracellular amyloid plaques, cerebrovascular amyloid deposits, intracellular neurofibrillary tangles, and neuronal loss are the pathological hallmarks of AD. The mechanisms underlying AD are not yet completely understood, but epidemiological and clinical studies suggest that an accumulation of misfolded proteins in the aging brain results in oxidative and inflammatory damage, which in turn leads to katabolic failure and synaptic dysfunction [3, 4].

There are several findings to support the hypothesis that the progressive production and subsequent aggregation of amyloid-β (Aβ), a 39-43 amino acid residue proteolytic fragment of the membrane-associated amyloid precursor protein (APP), is probably the key pathogenic event in the onset of AD. This link has motivated the search for therapies based on inhibition or reversal of Aβ aggregate formation. Different approaches have been proposed including blocking APP expression, reducing Aβ production, enhancing the clearance of Aβ, inhibiting the neurotoxic activity of Aβ and blocking the interaction of Aβ with amyloid-associated proteins [3-5].

**Fig. (1):**
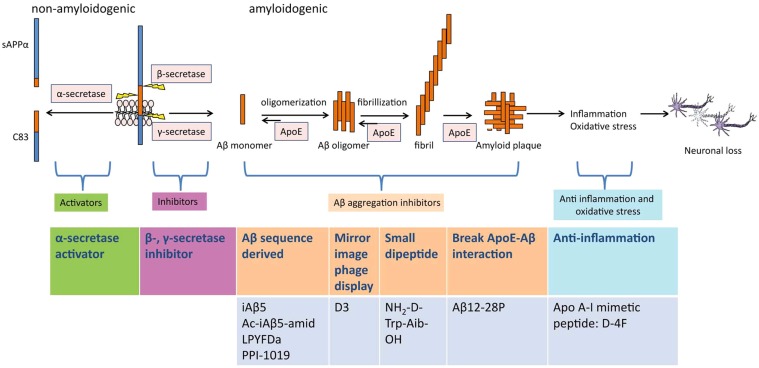
APP processing. The transmembrane protein APP can be processed along two main pathways, non-amyloidogenic pathway and amyloidogenic pathway. In the non-amyloidogenic pathway, α-secretase cleaves in the middle of the Aβ region and releases a soluble APP fragment (sAPPα) and a C83 carboxy-terminal fragment. In the amyloidogenic pathway, APP is sequentially cleaved by β-secretase and γ- secretase. Aβ is released and aggregates further to oligomers, fibrils and neurotoxic amyloid plaques. ApoE can act as a chaperone of Aβ, promoting its conformational transformation from soluble Aβ into toxic aggregates. Aβ plaques induce inflammatory responses and oxidative stress, and thereby cause neuronal loss. Strategies for therapeutic intervention in AD include α-secretase activation, β-/γ-secretase inhibition, Aβcleavage, anti-inflammation, anti-oxidative stress, *etc*. Several therapeutic peptides act as β-sheet breakers, anti-inflammation and anti-oxidative stress agents.Therapeutic peptides which have been studied both *in vitro* and *in vivo* are illustrated in the figure.

Aβ is generated by the cleavage of APP through the concerted actions of β- and γ-secretases. A third protease, α-secretase, which competes with β-secretase for the APP substrate, can preclude the production of Aβ (Fig. **1**). Therefore, specific inhibition of β- and γ-secretases and activation of α-secretase are important strategies for therapeutic intervention. While stimulations of these enzymes can effectively prevent the production of Aβ, these approaches are hampered by poor specificity and undesirable side effects. For example, the α-secretase pathway can be stimulated through cell-surface receptors. However, the desired reduction in Aβ requires a marked change in the metabolism of both APP and various other membrane proteins that are α-secretase substrates, which results in side effects [6, 7]. Similarly, the inhibition of γ-secretase causes subsequent problems on thymocyte differentiation with the inhibition of Notch cleavage [8, 9]. The deletion of β-secretase in mouse models results in an increased death rate and alterations in the steady-state inactivation of voltage-gated sodium channels [10]. Next to reduction of Aβ generation, A*β* immunization was considered as another promising strategy for AD therapy. However, a clinical study in humans has questioned this treatment by revealing that immunized patients showed a strong brain inflammatory response [11-13].

Alternatively, the inhibition of Aβ aggregation is another attractive therapeutic strategy because this appears to be the first step in the pathogenic process of amyloidosis. Several reports described different chemical compounds that blocked Aβ aggregation or dissolved amyloid fibrils [14-17]. Mc Laurin *et al*. demonstrated that naturally occurring inositol stereoisomers interacted with Aβ and stabilized small Aβ aggregates [18]. Pappolla *et al*. [19] reported that melatonin, a hormone that crosses the blood-brain barrier (bbb), can block Aβ fibril formation [20]. Beside the traditional small molecule drugs, therapeutic peptides emerged to be an attractive area for drug discovery because of their high biological activity associated with low toxicity and high specificity. The benefits conferred by these characteristics include little unspecific binding to molecular structures other than the desired target, minimization of drug-drug interactions and negligible accumulation in tissues, reducing risks of complications due to metabolic products or intermediates. In addition, these peptides and peptidomimetic molecules by themselves or with further modifications provide a class of reagents that may help to elucidate the mechanism of amyloid aggregation, perhaps by trapping intermediates, as well as providing inroads into the design of diagnostic and therapeutic reagents. A wide range of Aβ binding peptides, which block amyloid formation, were investigated to date for therapeutic purposes [21-25]. Only very few were shown to be effective in rodent AD models or in clinical studies (Table 1). Here, we review those peptides and discuss their possible mechanisms of action.

## 1 β-SHEET BREAKER PEPTIDES - iAβ5 AND ITS DERIVATIVES

**Fig. (2):**
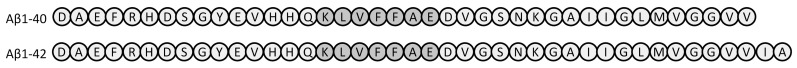
Illustration of KLVFFAE (Aβ(16-22)), SRE of Aβ. Aβ1-40 and Aβ1-42 are the major forms of Aβ peptides in the brain. Here, the sequences of both peptides are illustrated. The SRE part of Aβ, KLVFFAE, is indicated.

An attractive therapeutic strategy for the treatment of AD is to identify and to investigate so called β-sheet breaking compounds. β-sheet breaker peptides combine a sequence similarity to the region of the protein involved in the abnormal folding (self-recognition element (SRE)), but do not allow further elongation of β-sheet fibril structures (Fig. **2**) [23]. In 1996, Tjernberg *et al.* described that peptides incorporating a short Aβ fragment (KLVFF; Aβ16-20) can bind full-length Aβ and prevent its further assembly into amyloid fibrils. This represented a starting point for modified peptide aggregation inhibitors [22, 41]. Based on this finding Soto *et al*. designed an 11 amino acid peptide, called inhibitor of Aβ fibrillogenesis peptide 1 (iAβ1) (RDLPFFPVPID), which binds to Aβ and inhibits Aβ fibril formation *in vitro *[30]. Furthermore, a shorter anti-β-sheet peptide, iAβ5, was designed using iAβ1 as a model, which showed similar inhibitory activities but improved bbb permeability due to the reduced molecular mass [30].

iAβ5 is an inhibitor of Aβ fibrillogenesis with the sequence LPFFD [30]. The structure design of iAβ5 is based on the central hydrophobic region within the N-terminal domain of Aβ, amino acids 17-20 (LVFF) [42, 43]. As a substitution of valine (V), a proline (P) was included to decrease the peptide’s propensity to adopt a β-sheet structure. Additionally, the charged residue aspartate (D) was added at the end of the peptide to increase hydrophobicity and bbb permeability [23].

Despite its significant activity to inhibit Aβ fibrillogenesis, iAβ5 was unstable and easily degraded by proteases which hampered its therapeutic usage *in vivo *[27]. By adding charged sequences, polyethylene glycerol (PEG) [44], N-methylation [45], using D-enantiomeric residues, replacing residues with tyrosine, and introducing N-terminal acetylation and C-terminal amidation [26, 27], iAβ5 derivatives with enhanced properties, i.e. higher proteolysis resistance, stability, activity, solubility and bbb permeability could be established [28, 46].

iAβ5 and its derivatives (see Table **2**) have been studied extensively both *in vitro* and *in vivo*. Soto *et al.* demonstrated that iAβ5 can inhibit amyloid formation of Aβ1-40 and Aβ1-42 *in vitro*. Thioflavine T (ThT) fluorometric assays showed that iAβ5 induced a disassembly of preformed Aβ fibrils. Furthermore, iAβ5 significantly reduced Aβ induced toxicity in human neuroblastoma cell culture [23, 30]. As a molecular mechanism it was proposed that the β-sheet breaker peptide inhibited amyloid formation by binding to monomeric and/or dimeric Aβ peptides, thereby blocking the formation of the oligomeric β-sheet-conformation precursor of the fibrils. The disassembly of preformed fibrils induced by iAβ5 may indicate that monomeric Aβ was in equilibrium with Aβ fibrils, as previously suggested [47, 48]. A molar excess of the inhibitor may bind to monomeric peptide thus displacing the equilibrium, and leading to Aβ fibril disaggregation. Another possibility may be iAβ5 binding to Aβ fibrils leading to amyloid destabilization [23, 30].

*In vivo* studies using a Fischer-344 rat brain model of amyloidosis indicated that iAβ5 can significantly reduce cerebral Aβ deposition and completely block amyloid fibril formation *in vivo* [23, 29]. Intraperitoneal administration of Ac-iAβ5-amid on the rat with behavioral deficits, induced by the intrahippocampal injection of Aβ-fibrils, demonstrated neuroprotective effects. More importantly, following the iAβ5 treatment, hippocampal-dependent spatial learning paradigms, including the standard Morris water maze and a working memory analysis, showed a significant prevention from impairments induced by Aβ deposits in the dorsal hippocampus [26]. In 2002, Permanne *et al.* carried out *in vivo* studies of Ac-iAβ5-amid using AD transgenic mouse models. The results demonstrated that intraperitoneal injected iAβ5 elicits a significant increase in neuronal survival and a decrease in brain inflammation associated with the reduction of amyloid plaques [27]. Moreover, improved pharmacological properties and a high capability of bbb permeability were indicated [27].

## 2 LPYFDA

In 2004, Dakti *et al.* designed the pentapeptide LPYFDa on the basis of the iAβ5 peptide LPFFD. In order to increase binding affinities to Aβ peptides, two changes in the structure were performed: (1) one of the phenylalanines was replaced with tyrosine and (2) the C-terminal carboxylate group was amidated to increase the binding affinity according to quantum chemical calculations. The new peptide LPYFDn was proven to have a very high binding affinity to Aβ-fibrils [33]. LPYFDa was demonstrated to be an excellent inhibitor of Aβ1-42 aggregate dependent neurotoxicity and almost completely prevented Tau hyperphosphorylation caused by Aβ1-42. In the MTT (3-(4,5-dimethylthiazol-2-yl)-2,5-diphenyltetrazolium bromide) bioassay, a 5-fold molar excess of LPYFDa prevented the neurotoxic effect of Aβ1-42. Additionally, LPYFDa partly prevented the binding of Aβ1-42 clusters to SH-SY5Y cells and neurite degeneration [33].

Juhasz and Szegedi *et al. *performed electrophysiological studies and neuronal excitation tests for LPYFDa both *in vitro *and *in vivo *[31, 32]. *In vitro* electrophysiological experiments on rat brain slices demonstrated that LPYFDa counteracted with the field excitatory postsynaptic potential-attenuating effect of Aβ1-42; *in vivo* experiments using extra cellular single-unit recordings combined with iontophoresis revealed that LPYFDa protected neurons from the NMDA response-enhancing effect of Aβ1-42 in the hippocampal CA1 region [32]. Further investigations using *in vivo *biodistribution of tritium-labelled LPYFDa and single-unit electrophysiology showed the ability of LPYFDa to cross the BBB and protect the synapses against the excitatory action of fibrillar Aβ [31]. LPYFDa may serve as a lead compound for the design of novel drug candidates for the prevention of AD.

## 3 PPI-1019

**Fig. (3):**
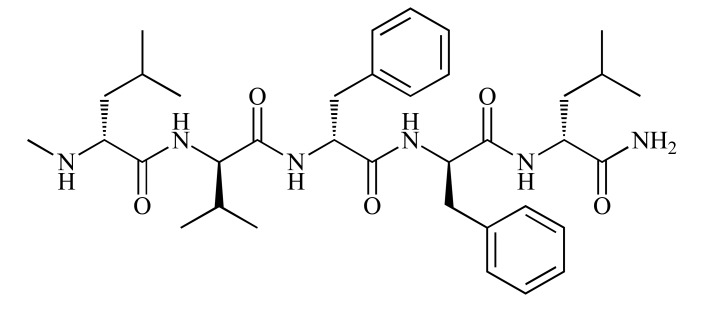
Chemical structure of PPI-1019. The sequence of PPI- 1019 is D-methyl-LVFFL [34, 35].

N-methylation is a generally used strategy to generate inhibitors of amyloidosis. On one hand, replacement of the hydrogen attached to the amide nitrogen with an alkyl group can prevent endopeptidase degradation [49, 50], on the other hand, N-methyl groups in place of backbone amide groups present a blocking face thereby is constrained to a β-sheet conformation [46, 51]. Based on the Aβ sequence 17-20 (LVFF), and after rounds of optimization, the N-methyl derivative PPI-1019 (methyl-LVFFL, D-form) (Fig. **3**), was demonstrated to have optimal properties to inhibit Aβ aggregation. The fundamental difference of PPI-1019 as compared to other N-methylated peptides is that an amine is methylated rather than an amide. A singly methylated amine still has two hydrogen-bond donors, therefore the mechanism of action may well be different from peptides having methylated amides, where hydrogen-bond donation is eliminated [51]. PPI-1019 has completed phase I and II human clinical trials, but further information has not been revealed [34, 35].

## 4 A DIPEPTIDE AS A (-SHEET BREAKER

In 2002, Gazit and coworkers demonstrated the key role of aromatic residues in amyloid formation [52, 53] and later on suggested a new direction for the development of amyloid formation inhibitors by using an Aib moiety as a β-breaker, in addition to aromatic recognition motifs [54]. Based on these findings, they developed a small D-enantiomeric dipeptide inhibitor of Aβ aggregate formation, named NH_2_-D-Trp-Aib-OH [36].

**Fig. (4):**
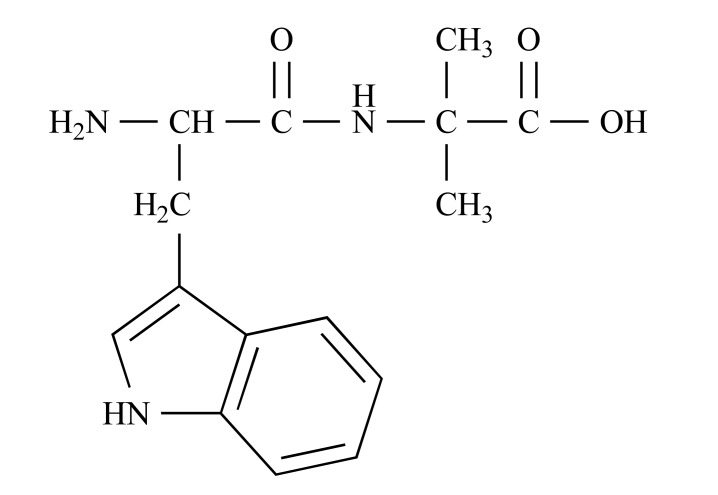
The chemical structure of NH_2_-D-Trp-Aib-OH [36].

NH_2_-D-Trp-Aib-OH was designed by performing iterative selection cycles on a library that combined aromatic recognition motifs and β-sheet breaker motifs. It combined an indole, which was identified as a potent aromatic binder of Aβ, and α-aminoisobutyric acid (Aib), which inhibited Aβ assembly into toxic oligomers by a C(alpha)-methylation beta-breakage strategy. Aib is a unique β-sheet breaker. The achiral amino acid has two methyl residues attached to the C_α_ atom and strongly favors helical conformations. The effect of Aib incorporation on the conformation of short peptides has been studied extensively over the last two decades. The Aib residue has been shown to have a very high tendency to induce helical conformations and to disrupt β-sheet structures in a large number of peptides. NH_2_-D-Trp-Aib-OH contained metabolically stable D-amino acids to increase its potential as a drug lead (Fig. **4**). Although the compound was designed initially by the principles of peptide chemistry, it overcomes many limitations of previous peptide-based amyloid inhibitors. By virtue of its physicochemical and pharmacokinetic properties, it has many characteristics of an ideal small-molecule drug, with a molecular weight of 289 Da, high serum stability, oral bioavailability, low toxicity, high solubility, and chemical stability in solution [54-58].

*In vitro* and *in vivo* experiments were carried out to investigate the inhibition efficiency and therapeutic potency of NH_2_-D-Trp-Aib-OH. SDS-PAGE, fluorescence anisotropy, and transmission electron microscopy experiments proved that the small-molecule inhibitor interacted with early intermediate assemblies of Aβ and inhibited their assembly into toxic oligomers. NMR spectroscopy data suggested that the inhibitor interacted specifically with the aromatic core of Aβ. The percentage of the compound that crossed the bbb was in the range of 4 to 8%. The orally bioavailable compound reduced the amount of amyloid deposits in the brain of AD model mice. Treatment with this compound led to the recovery of the cognitive performance of model mice to the level of nontransgenic mice. The ability of the compound to restore cognitive performance in AD transgenic mice further suggested that the targeting of early oligomers is a promising strategy for the treatment of AD [36].

## 5 BLOCKING OF A(-APOE4 INTERACTION BY THE PEPTIDE A(12-28P 

A major genetic risk factor for sporadic AD is the apolipoprotein (apo) E4 allele [59, 60]. The apoE4 protein strongly associates with enhanced vascular amyloid and plaque amyloid deposits of Aβ in the brains of AD patients [61]. ApoE was immunochemically localized in the senile plaques, vascular amyloid, and neurofibrillary tangles of AD. ApoE in cerebrospinal fluid was proven to bind to synthetic Aβ peptide with high avidity *in vitro *[60]. Pathologically, ApoE was described to act as a chaperone of Aβ, promoting its conformational transformation from soluble Aβ into toxic aggregates [37]. Therefore, therapeutic strategies were designed based on the blocking of apoE/Aβ interaction and thereby predicted to reduce Aβ toxic aggregates. In 2004, Sadowski *et al. *reported an Aβ-ApoE4 interaction blocking peptide, Aβ12-28P, and performed extensive investigations both *in vitro* and *in vivo*.

**Fig. (5):**
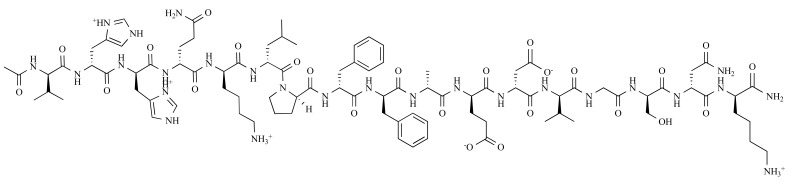
Chemical structure of Ac-Aβ12-28P-Amid [37].

The binding site of ApoE on Aβ corresponds to residues 12 to 28 [60, 62]. This sequence encompasses a hydrophobic domain (residues 14-21) and a β-turn (residues 22-28) which place two hydrophobic domains of Aβ (14–21 and 29–40/42) opposite each other allowing the assembly of Aβ peptides into fibrils [63]. Therefore, Aβ12-28P (Ac-VHHQKLPFFA EDVGSNK-Amid, D-enantiomeric form) (Fig. **5**), which functioned as a competitive inhibitor of the binding of full-length Aβ to ApoE, was designed to block the apoE/Aβ interaction. The substitution of valine at position 18 to proline changed the peptide's properties, making it non-fibrillogenic and non-toxic. Additionally, the use of D-enantiomeric amino acids, amidation of the C-terminus, and acetylation of the N-terminus minimized protease degradation and extended the serum half-life of the peptide to 62 minutes, in contrasting to the very short half-life of L-amino acid, non-end-protected Aβ1–40 (2 to 3 minutes) [37, 64].

To analyze the inhibition effect of Aβ12-28P on Aβ fibrillogenesis and toxicity, a series of *in vitro* experiments were conducted. A competitive inhibition assay indicated that Aβ12-28P competitively blocked binding of full-length Aβ to apoE and reduced Aβ fibrillogenesis. The effect of apoE on Aβ fibril formation and toxicity in cell culture was significantly reduced in the presence of Aβ12–28P. Taken these results together, Aβ12-28 was proven to be able to inhibit Aβ/apoE interaction and thereby blocked apoE pathological chaperoning properties on Aβ fibrillogenesis and toxicity. Moreover, Aβ12-28P was bbb permeable allowing for an *in vivo *effect within the brain. APP/PS1 double transgenic mice treatment with Aβ12-28P for 1 month had resulted in a 60% reduction of Aβ load in the cortex and in the hippocampus comparing to the control groups. These findings indicated the inhibition effect of Aβ12-28P of Aβ deposition *in vivo* and demonstrated that compounds blocking the interaction between Aβ and its pathological chaperone ApoE may be beneficial for treatment of Aβ deposition in AD [37]. Further exploration including biochemical and behavioral studies of treatment affects in AD transgenic models is needed.

## 6 APOA-I MIMETIC PEPTIDE - D-4F

Beside the well investigated Aβ amyloid plaques, epidemiological and clinical studies suggest that inflammation and oxidative stress are implicated in AD [65-68]. However, the role of inflammation in the pathogenesis of AD is not clearly understood [38]. Hypercholesterolemia is an important risk factor in the development of AD. Emerging evidence suggests that high dietary cholesterol increases Aβ accumulation and accelerates AD-related pathology [69, 70]. ApoA-I, the major protein component of High-density lipoprotein (HDL), has a central role in reverse cholesterol transport [71, 72], anti-oxidant and anti-inflammatory properties [73].

The apoA-I mimetic peptide, D-4F, was developed based on the presence of lipid-associating amphipathic α-helices in ApoA-I and possessed the ability to avidly bind lipids [74, 75]. Notably, ApoA-I and derivatives thereof have indeed been used to prepare self-assembled proteolipid particles, termed nanodiscs, for solubilization of membrane attached proteins and even integral membrane proteins within flat small pieces of lipid bilayers render them applicable to for biophysical methods reserved to fully water soluble molecules [76-81].

**Fig. (6):**
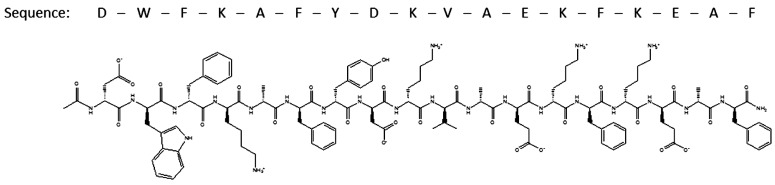
The chemical structure of D-4F [86].

D-4F is synthesized from D-amino acids with the sequence Ac-DWFKAFYDKVAEKFKEAF-NH_2_ (Fig. **6**). It possesses a class A amphipathic helical structure with opposing polar and nonpolar faces which is responsible for lipid association [74]. The structure of D-4F was initially taken from the peptide 18A (DWLKAFYDKV AEKLKEAF), which does not have any sequence homology to apoA-I but does form a class A amphipathic helix that has some of the lipid-binding properties of apoA-I. Replacing both leucines (L) with phenylalanines (F) and adding protections of the N- and C- termini in 18A by acetyl and amid groups respectively, yielded D-4F (named 4F because it contains four phenylalanine residues), which showed increased helicity, self-association and lipid affinity. The molecular volume of the L and F residues differs significantly. L possesses an aliphatic side chain and is able to interdigitate with lipid acyl chains. F has an aromatic side chain and, compared with L, has a larger nonpolar accessible surface area [82]. These two properties allow the D-4F to reside close to lipid head groups, positioning the delocalized π-electron system in the hydrophobic milieu to allow for the sequestration of water or polar lipid hydroperoxides in the membrane [75, 83].

Most studies of D-4F were focused on its potential role in atherosclerosis management [84, 85]. It has been shown that oral administration of D-4F, synthesized from D-amino acids, remained intact in the circulation, significantly enhanced HDL protective capacity, decreased low density lipoprotein (LDL) induced monocyte chemotactic activity, and inhibited the formation of atherosclerotic plaques in young apoE null mice [86]. Oral D-4F synergized with pravastatin increased intestinal apoA-I synthesis and plasma apoA-I levels, and caused lesion regression in old apoE-null mice [87].

Several studies suggested that AD may have an inflammatory component similar to atherosclerosis that is associated with very small vessels such as arterioles [69, 88, 89]. In 2009, Handattu *et al. *reported the first time D-4F being used for oral therapies in a mouse model of AD. They performed *in vitro* and *in vivo *experiments to evaluate the efficacy of oral D-4F co-administered with pravastatin on cognitive function and Aβ burden in the hippocampus of APPSwe-PS1 Delta E9 mice. Behavior tests showed significant improvement in cognitive function for the D-4F administered animals. Furthermore, oral D-4F significantly reduced Aβ load in the hippocampal region of the brain. Moreover, there was a significant decrease in the number of activated microglia and activated astrocytes upon oral D-4F treatment. Inflammatory markers interleukin-1β, tumor necrosis factor-α levels, and the expression of monocyte chemoattractant protein-1 were decreased significantly in the D-4F group. These results suggest that the D-4F inhibits amyloid beta deposition and improves cognitive function via exerting anti-inflammatory properties in the brain [38].

## 7 D3 SELECTED USING MIRROR IMAGE PHAGE DISPLAY

Recently, D3, a highly specific ligand for Aβ, has been identified using a mirror image phage display approach with a huge randomized 12-mer peptide library (> 1 billion different peptides). Mirror phage display allows the use of phage display to identify peptides that consist solely of D-amino acids and bind specifically to a given target [90]. Compared with L-peptides, D-amino-acids are less protease sensitive, more resistant to degradation in animals and less or even not at all immunogenic. Briefly, the Aβ1-42 D-enantiomer was synthesized, which is the exact mirror image of the naturally occurring L-Aβ1-42. Then, the D-Aβ 1-42 was used as a target for selection of peptides from a 12-mer randomized peptide library displayed on the surface of M13 bacteriophages for those that bind best to D-Aβ1-42. For reasons of symmetry, the D-enantiomeric form of the selected 12-mer peptide will also bind to the nature L-form of Aβ1-42 [91]. Using this strategy, the dominant peptide sequence RPRTRLHTHRNR was obtained, referred to as D3 consisting of D-enantiomeric amino acids, which showed high binding affinity and specificity to Aβ. The structure of D3 is illustrated in Fig. (**7**). Extensive *in vivo* and *in vitro* experiments have been performed indicating the promising therapeutic potential of D3 for AD treatment [39, 40, 92].

To elucidate the possible mechanism of action of D3 on Aβ, several *in vitro* experiments were performed. Funke *et al. *carried out *in vitro* investigations, using dynamic light scattering, turbidity measurements, density gradient centrifugation analysis and size exclusion chromatography of Aβ/D3 mixtures. D3 was shown to abolish Aβ oligomers and induce the formation of large Aβ aggregates. The large-sized Aβ aggregates induced by D3 showed neither a positive ThT signal nor amyloid properties upon staining with Congo red, indicating the absence of regular fibrils [40]. Consistent with the experimental observation of large nonfibrillar Aβ aggregates in the presence of D3, further computational simulations of an Aβ nonamer in the presence and absence of D3 proved strong interactions between the arginine-rich D3 and negatively charged groups of Aβ, which were expected to compensate the charge on the Aβ surface and reduce solubility and promote the aggregation of Aβ. Moreover, D3 binding also showed effects on the topology of the Aβ oligomers, which induced a large twist and facilitated the formation of nonfibrillar aggregates [40]. Taken together, the *in vitro* data clearly showed that D3 is able to precipitate toxic Aβ oligomers into large, high-molecular-weight, nontoxic, ThT negative, nonamyloidogenic amorphous aggregates that fail to act as seeds in Aβ fibril formationassays. In all assays, D3 did not increase the concentration of monomeric Aβ. In 2009, van Groen *et al. *demonstrated the usage of FITC-labelled D3 for both *in vitro* and *in vivo* staining of Aβ-1-42 in the brains of transgenic AD-model mice [39]. Additionally, D3 was proven to have good bbb permeability in an *in vitro* bbb cell culture model which further demonstrated the therapeutic potential of D3 [93]. *In vivo*, D3 reduced plaque load and cerebral inflammation of AD transgenic mice [92]. Most recently, Funke *et al*. carried out oral treatment with D3 to AD transgenic mice and performed behavior studies. Significant cognitive improvement and reduction of plaque-related inflammation were detected [40]. These data demonstrated that the therapeutic potential of D3 in model mice might be related to the potential mechanism that D3 precipitates toxic Aβ oligomers *in vitro* and converts them into nonamyloidogenic, nonfibrillar, and nontoxic aggregates. One possible mode of D3 action may be that the D3-induced conversion of Aβ species into amorphous Aβ-D3 aggregates adds an additional equilibrium to the complex network between the various Aβ species. D3 thereby shifts the equilibria among Aβ monomers, oligomers, and fibrils toward Aβ-D3 aggregates that are nonamyloidogenic and may be more amenable to degradation processes [40]. In 2010, Müller-Schiffmann *et al.* reported on the D3 hybrid compound JM169, which combined the D-enantiomeric peptide with a β-sheet breaking compound via a linker substance. The authors demonstrated that chemical synthesis of two entirely different substance classes acting on the same target can be covalently linked to yield dramatic synergistic effects and lead to novel properties [94].

## CONCLUSION

Based on the amyloid hypothesis, the progressive accumulation of Aβ is considered as a primary driver of AD-related pathogenesis, including neurofibrillary tangle formation, synapse loss and neuronal cell death [95]. Therefore, promising therapeutic strategies of AD are the application of neuroprotective agents that selectively inhibit Aβ aggregation and/or enhance clearance of Aβ peptides and amyloid deposits.

Beside the traditional organic compounds that have been reported to inhibit or reduce the aggregation and toxicity of Aβ [14-17], peptide inhibitors of Aβ aggregation emerge to be alternative therapeutic agents for AD, because of their high specificity, low toxicity and high biological activity [96, 97]. However, due to the biological properties, therapeutic peptides have weak points, like immunogenicity, low stability, low solubility, poor bioavailability and low bbb permeability [96]. To overcome these shortages and improve the molecules’ properties, suitable chemical modifications, including N- or C-terminal modifications, incorporation of conformationally constrained amino acids, or modifications of the peptide backbone, have been performed. Moreover, N-terminal acetylation and C-terminal amidylation is also common used to protect peptides against proteolytic degradation and increased the bbb permeability [26-28]. Beside the chemical modifications, another promising strategy to improve the peptide stability is the usage of D-enantiomers which are considered to be rather protease resistant [24, 28, 98, 99] and mostly nonimmunogenic [90, 99, 100]. However, chemical modification of peptides with therapeutic properties may have drawbacks such as activity loss and toxicity [28]. Therefore, *in vivo* and *in vivo* experiments should be carefully designed to obtain proper chemical modifications which do not alter the peptide’s ability to prevent fibrillogenesis, but significantly increases its bbb permeability and resistance to proteolysis.

In this review we summarized the peptide inhibitors of Aβ which showed significant therapeutic effects in AD animal models and/or human clinical trials. Special structure features of these therapeutic peptides were described, including SRE analog sequence, arginine-rich sequence, aromatic residue and amphipathic helical structure. These properties facilitate their binding to Aβ and amyloid associated proteins, and thereby block amyloid formation. Proline residues are widely used since it has greater β-breaking potential than the other proteinogenic amino acids [30, 101]. Another approach involves N-methylation of peptide inhibitors that prevent β-sheet stacking by interfering with the intermolecular backbone hydrogen bonds needed to form the structure [46, 51]. This provides an avenue for designing diagnostic and therapeutic reagents for AD.

For a long time, it was assumed that Aβ assembled into extracellular amyloid fibrils and plaques resembles major pathogenic species in AD. However, over the past decade, accumulating evidence suggests that soluble forms of Aβ are the proximate effectors of synapse loss and neuronal injury [102]. Thus, preventing the formation of cytotoxic oligomers should prove an effective means for treating AD. The targeting of early molecular-recognition and self-assembly processes appears to be more promising for the treatment of AD than the disassembly of formed mature amyloid fibrils. According to these hypotheses, D3 and NH_2_-D-Trp-Aib-OH can be predicted to be attractive therapeutic agents for AD [36, 40].

Taken together, the strategies of designing and modifying therapeutic peptides to improve their activity and drug-like properties offer a general solution to discover peptide inhibitors for Aβ aggregation and toxicity. These efforts provide further understanding of the mechanism of Aβ aggregation. However, due to the complex pathophysiology of AD, since extensive in *vivo *and* in vitro* investigations were performed, accurate interpretations of the mechanism of actions remain problematic. Future research to translate these peptides and peptidomimetics to drug in treatment of AD need to be established.

## Figures and Tables

**Fig. (7):**
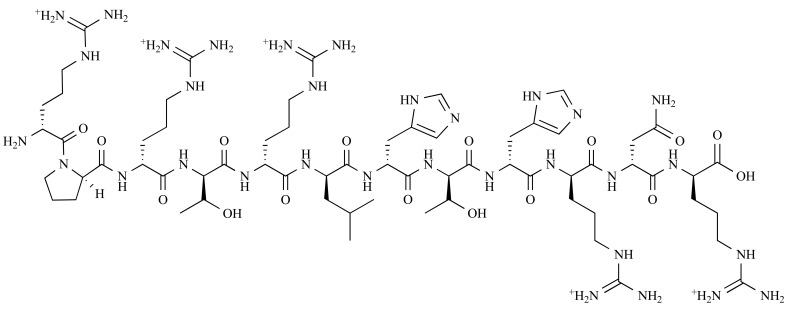
Chemical structure of D3 [40].

**Table 1.** AD Therapeutic Peptides that Were Shown to be Effective in Rodent AD Models or in Clinical Studies. The Peptide Category, Name, Sequence, Description and Related References are Indicated in the Table

Category	Name	Sequence	Description	References
Aβ-sequence derived β-sheet breaker peptides	iAβ5	LPFFD	Proline based β-sheet breaker	[23, 26-30]
	Ac-iAβ5-amid	Ac-LPFFD-amid	iAβ5 derivatives to improve pharmaceutical properties	[26, 27]
	LPYFDa	LPYFDamid		[31-33]
	PPI-1019	Methyl-LVFFL	Completed phase II clinical trial	[34, 35]
Dipeptide β-sheet breaker	NH_2_-D-Trp-Aib-OH	Ac-Trp-Aib	β-sheet breaker	[36]
Aβ-ApoE4 interaction blocker	Aβ12-28P	Ac-VHHQKLPFFAEDVGSNK-Amid	Aβ-sequence derived	[37]
Anti-inflammation and oxidative stress compound	D-4F	Ac-DWFKAFYDKVAEKFKEAF-NH_2_	Apo A-I mimetic peptide	[38]
Selected with combinatorial peptide libraries	D3	RPRTRLHTHRNR	Mirror image phage display of combinatorial peptide libraries	[40]

**Table 2.** Chemical Structures of KLVFF, iAβ5 and its Derivatives. The Chemical Structures and Enantiomeric forms of KLVFF, iAβ5 and its Derivatives are Illustrated in the Table. The Peptide Analogs Containing D-amino Acids are more Resistant to Proteolytic Degradation.

Name	Structure	D/L	References
KLVFF	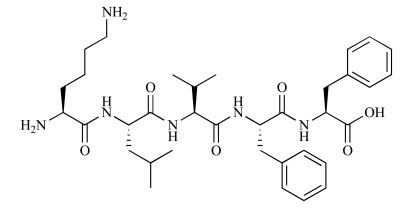	L	[22, 41]
iAβ5: LPFFD	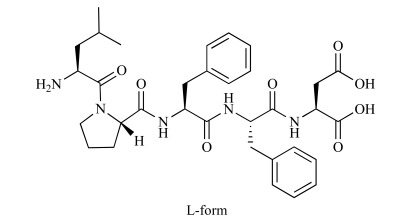	L/D	[23, 30]
Ac-iAβ5-amid	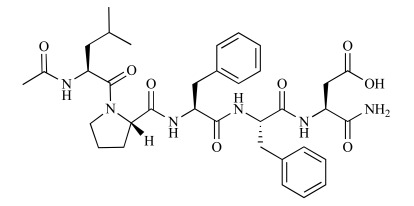	L	[26, 27]
LPYFDa	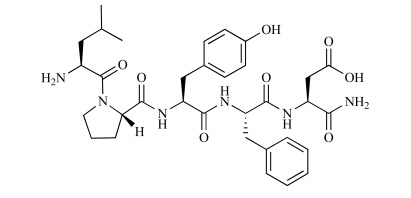	L	[31, 33]

## ABBREVIATIONS

AD: = Alzheimer’s disease
Aib: = α-Aminoisobutyric acid
Aβ: = amyloid β
APP: = amyloid precursor protein
Apo: = apolipoprotein
bbb: = blood-brain barrier
F: = phenylalanine
fluo-D3: = fluorescein labeled D3
HDL: = High-density lipoprotein
iAb: = inhibitor of Aβ fibrillogenesis peptide 1
L: = leucine
LDL: = low density lipoprotein
PEG: = polyethylene glycerol
sAPPα: = soluble APP fragment
SRE: = self-recognition element
ThT: = Thioflavine T
